# Cell wall traits as potential resources to improve resistance of durum wheat against *Fusarium graminearum*

**DOI:** 10.1186/s12870-014-0369-1

**Published:** 2015-01-19

**Authors:** Vincenzo Lionetti, Angelica Giancaspro, Eleonora Fabri, Stefania L Giove, Nathan Reem, Olga A Zabotina, Antonio Blanco, Agata Gadaleta, Daniela Bellincampi

**Affiliations:** Dipartimento di Biologia e Biotecnologie “Charles Darwin”, Sapienza Università di Roma, Rome, Italy; Department of Soil, Plant and Food Science (DiSSPA), University of Bari “Aldo Moro”, Via G. Amendola 165/A - 70126, Bari, Italy; Roy J. Carver Department of Biochemistry, Biophysics and Molecular Biology, Iowa State University, Ames, Iowa 50011 USA

**Keywords:** Fusarium Head Blight resistance, Wheat, Pectin methylesterase, Cell wall, *Fusarium graminearum*

## Abstract

**Background:**

*Fusarium graminearum*, one of the causal agents of Fusarium Head Blight (FHB, scab), leads to severe losses in grain yield and quality due to the production of mycotoxins which are harmful to human and livestock. Different traits for FHB resistance in wheat were identified for common wheat (*Triticum aestivum L.*) while the sources of FHB resistance in durum wheat (*Triticum turgidum* ssp. Durum), one of the cereals most susceptible to *F. graminearum* infection, have not been found. New lines of evidence indicate that content and composition of cell wall polymers affect the susceptibility of the wall to degrading enzymes produced by pathogens during infection and can play a role in the outcome of host-pathogen interactions. The objective of our research is to identify potential cell wall biochemical traits linked to *Fusariosis* resistance to be transferred from a resistant common wheat to a susceptible durum wheat line.

**Results:**

A detailed analysis of cell wall composition in spikes isolated from a highly resistant common wheat accession “02-5B-318”, a breeding line derived from the FHB-resistant Chinese cv. Sumai-3 and a high susceptible durum wheat cv. Saragolla was performed. Significant differences in lignin monolignols composition, arabinoxylan (AX) substitutions and pectin methylesterification were found between resistant and susceptible plants. We isolated and characterized a pectin methylesterase gene *WheatPME1,* which we found being down regulated in the FHB-resistant line and induced by fungal infection in the susceptible wheat.

**Conclusions:**

Our results indicate cell wall traits differing between the FHB sensitive and resistant wheat genotypes, possibly related to FHB-resistance, and identify the line 02-5B-318_R_ as a potential resource of such traits. Evidence suggests that *WheatPME1* is involved in wheat response to *F. graminearum.*

**Electronic supplementary material:**

The online version of this article (doi:10.1186/s12870-014-0369-1) contains supplementary material, which is available to authorized users.

## Background

Durum wheat (*Triticum turgidum ssp. durum*) and common wheat (*Triticum aestivum L.*) are largely cultivated in European countries and the grain used for the human alimentation (http://www.FAO.org) and animal feeds. Common wheat allows producing wheat flour and bread, while durum wheat is primarily processed into semolina to produce pasta and couscous and some specialty breads. *Fusarium graminearum*, one of the major global pathogens of cereals, is considered the main causal agent of Fusarium head blight (FHB) disease in wheat [1]. *F. graminearum* infection causes a significant grain yield and quality loss by producing trichothecene mycotoxins that make harvest unsuitable for human and animal consumption [2]. Host resistance is the primary trait used as a control measure, and its manipulation is the best economic and ecological strategy to reduce damage caused by FHB disease. However, the molecular bases of wheat resistance and susceptibility to *F.graminerum* are scarcely known [3]. Resistance to FHB is a complex and quantitative trait controlled by multiple genes and characterized by large genetic variation in wheat gene pool [4]. Several studies aimed to identify traits involved in FHB resistance were carried out using common wheat (*Triticum aestivum L.*) while limited information is available for durum wheat (*Triticum turgidum* ssp. Durum), which is currently one of the cereals most susceptible to *F.graminearum* infection [4]. Even though in the last decade different studies were focused on the identification of candidate genes involved in *F.graminerum* resistance in cultivated or wild durum germoplasm, to date the sources of FHB resistance in durum wheat have not been fully identified [4-7].

*F. graminearum* preferentially infects wheat spikelets at the stage of anthesis, performs inter and intra-cellular growth and spreads systemically along the rachis [2]. During infection, *F. graminearum* produces cell wall degrading enzymes (CWDEs), such as pectinases, xylanases and cellulases, to degrade cell wall polysaccharides to penetrate and colonize the host tissues [8-10]. The role of cell wall components in plant resistance to disease has been scarcely studied in grasses. New lines of evidence indicate that content and composition of cell wall polymers affect the susceptibility of cell wall (CW) to CWDEs and can play a role in the outcome of host-pathogen interactions [11-14]. Notably, the extent of CW degradation is often associated with severity of disease [15] Cell wall polysaccharides of the graminaceous monocots (Type II cell wall), consist of a network of cellulose fibers embedded in a matrix of hemicelluloses, such as arabinoxylan (AX) and mixed linkage glucans (MLG), with a minor amount of xyloglucan and pectins [16]. AX (20-40% of CW dry weight) is composed of a β1,4-linked xylose backbone substituted by different monosaccharides, such as arabinose, glucuronic acid and, to lesser extent, galactose [17]. The degree of arabinose substitutions are thought to affect the AX degradability by fungal xylanases [18]. MLGs (10-30%) is an unbranched polysaccharide consisting of blocks of (1,4)-β-linked D-glucose residues interrupted by single (1,3)-β-linkages [16,19]. Pectins (5-10%) are complex polymers with different structural domains including homogalacturonan (HG), rhamnogalacturonan I (RG-I), rhamnogalacturonan II (RG-II) and xylogalacturonan (XG). Galacturonosyl residues of pectin backbones are methylesterified in Golgi apparatus and secreted into the cell wall in a highly methylesterified form. In the apoplasm, pectins are de-methylesterified by pectin methyl esterases (PMEs), which modulate the degree and patterns of methylesterification [20]. The de-methylesterification of pectin affects its interaction with cellulose [21,22] and the formation of crosslinks between pectin chains and xyloglucan or lignin [23,24]. The methylesterification makes pectin less susceptible to degradation by pectin degrading enzymes produced by fungal pathogens [5,25-28]. Pectin content and methylesterification in grasses has been associated with plant resistance to pathogens [5,11,20,29,30]. Lignin is a complex aromatic heteropolymer comprising a substantial portion (20%) of the grasses cell wall. Lignin of monocotyledonous species includes three types of monomers such as p-hydroxyphenyl (H), guaiacyl (G), and syringyl (S) phenylpropanoid monolignols [31,32]. Lignin is an important structural component involved in defense against invasive pathogens, making the cell wall more resistant to CWDEs and also preventing the diffusion of the pathogen-produced toxins [33].

The objective of our research is to identify cell wall biochemical traits useful to improve FHB resistance in durum wheat. To that end, detailed comparative analyses of cell wall composition in spikes isolated from a highly resistant common wheat accession “02-5B-318”, a breeding line derived from the FHB-resistant Chinese cv. Sumai-3 and a highly susceptible durum wheat cv. Saragolla were performed. Significant differences in lignin composition, AX substitution and pectin methylesterification were found between resistant and susceptible plants. The genomic sequence and the chromosome location of *WheatPME1* gene, differently expressed in resistant and susceptible lines during *F. graminearum* infection and possibly involved in susceptibility to *Fusarium graminearum,* was identified and characterized.

## Results and discussion

### Assessment of *Fusarium* symptoms on wheat spikes

**Figure 1:**
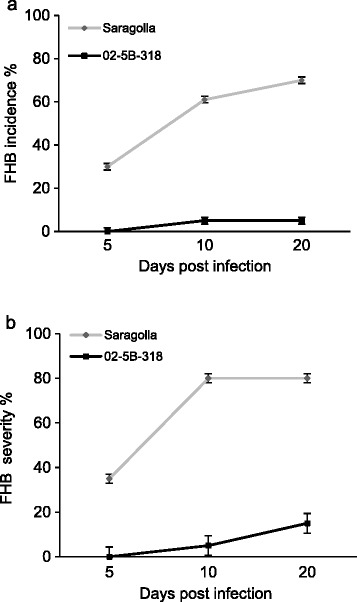
**Time-course analysis of FHB symptoms development following** ***F. graminearum*** **infection. (a)** FHB incidence and **(b)** FHB severity of Saragolla_S_ and 02-5B-318_R_ were evaluated. Data are the average ± standard deviation of two independent experiments (n ≥ 20). The average values of Saragolla_S_ and 02-5B-318_R_ lines are significantly different according to Student’s t test (*p* < 0.001).

In the present study, the resistance to FHB was analyzed in common wheat accession line 02-5B-318 and in Saragolla, known as one of the most susceptible durum wheat cultivar [34]. Spikes at anthesis were inoculated with fungal spores and disease symptoms were recorded 4, 10 and 20 days post-infection. Symptoms were evaluated as FHB incidence, expressed as percentage of infected spikes per genotype and FHB severity, expressed as percentage of spikelets showing symptoms on the total number of spikelets per spike [35]. Significantly higher FHB incidence and severity were observed in Saragolla (henceforth Saragolla_S_) in comparison with line 02-5B-318 (henceforth 02-5B-318_R_) (Figure 1a and b) indicating that the two genotypes exhibited quite extreme phenotypes for FHB resistance/tolerance.

### The cell wall of 02-5B-318_R_ spikes contain higher content of S lignin with respect to Saragolla_S_

A detailed analysis of the main structural cell wall components was performed in spikes of 02-5B-318_R_ and Saragolla_S_ plants, at anthesis. The characterization of lignin content and composition demonstrated that, while the two genotypes did not differ in the content of lignin, they showed significant differences in monolignols (Table 1). In particular, lignin of 02-5B-318_R_ spikes contained a significant higher percentage of syringyl (S) and p-hydroxyphenyl (H) monolignols and a lower amount of guaiacyl (G) monolignols, hence having a higher S/G ratio in comparison with Saragolla_S_ genotype. Recent studies aimed to elucidate the effects of lignin composition on the resistance of cell wall to degradation by decay fungi demonstrated that poplar lines extremely rich in syringyl lignin were recalcitrant to fungal degradation [36]. The transcript level of the cinnamoyl-CoA reductase CsCCR4 in the oilseed crop *Camelina sativa* was observed to be more than 10 times higher in the lines with the higher resistance to *Sclerotinia sclerotiorum* than in susceptible lines, and this correlated with an high level of constitutive S-lignin [37]. Suppression of F5H (ferulate/coniferaldehyde 5-hydroxylase) or CAOMT (caffeic acid O-methyltransferase), which reside on a branch pathway converting G to S monolignols, greatly reduced the S/G ratio [38]. In addition, the silencing of CAOMT in *Triticum monococcum* enhanced powdery mildew penetration [39]. Also, the synapyl alcohol-specific peroxidases involved in polymerization of monolignols can be regulated during *Fusarium* infection. Overall these results suggest that a higher S lignin content is a possible cell wall biochemical trait related to *Fusarium* resistance and also propose that genes favoring S-type lignin accumulation might potentially be involved in the resistance to the pathogen.

**Table 1:** **Lignin content and monolignol composition in cell walls from spikes of 02-5B-318**_**R**_ **and Saragolla**_**S**_ **plants**

	**02-5B-318**_**R**_	**Saragolla**_**S**_
Lignin (%)	10.65 ± 1.52	11.23 ± 2.27
S (%)	7.28 ± 0.91	**2.36 ± 1.00**
H (%)	30.65 ± 1.71	**20.86 ± 2.68**
G (%)	60.24 ± 4.33	**76.68 ± 2.11**
S/G ratio	0.121 ± 0.02	**0.031 ± 0.01**

Numbers in bold indicate statistically significant differences in each monolignol between the two genotypes, according to Student’s t-test (*p <*0.05).

### Xylans in cell wall of 02-5B-318_R_ spikes present a higher degree of arabinosylation with respect to Saragolla_S_

**Figure 2:**
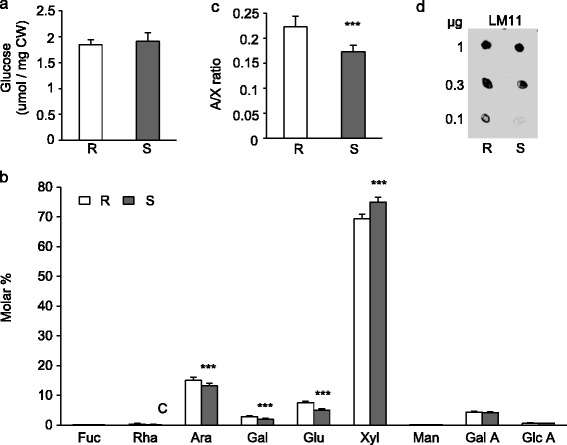
**Monosaccharide compositions and immunodot analysis of cell wall polysaccharides in spikes of 02-5B-318**_**R**_ **and Saragolla**_**S**_ **plants. (a)** Cellulose-derived glucose, **(b)** Fucose (Fuc), rhamnose (Rha), arabinose (Ara), galactose (Gal), glucose (Glc), xylose (Xyl), galacturonic acid (Gal A) and glucuronic acid (Glu A) released after 2 M TFA hydrolysis were determined by using a high-performance anion-exchange chromatography with pulsed amperometric detection (HPAEC-PAD) system, **(c)** Arabinose/Xylose ratio in spikes of 02-5B-318_R_ and Saragolla_S_. Results represent the mean ± SD of three replicates (n = 6). Asterisks indicate data sets significantly different between 02-5B-318_R_ and Saragolla_S_ according to Student’s *t*-test (*p <* 0.001). **(d)** Immunodot analysis for xylan substitution using LM11 antibody. The micrograms of in KOH 1 M hemicellulose fraction from the two genotypes were applied to the nitrocellulose membrane were indicated. The experiments were repeated three times with similar results. R = 02-5B-318_R_; S = Saragolla_S_.

We performed a comparative analysis of CW polysaccharides of 02-5B-318_R_ and Saragolla_S_ wheat plants. The cell walls were extracted from spikes and the cellulose content as well as monosaccharide composition of the non-cellulosic polysaccharides were determined (Figure 2). The amount of the cellulose-derived glucose was not significantly different between the two genotypes indicating that cellulose content is not related to their different FHB resistance/susceptibility (Figure 2a). Monosaccharide composition of non-cellulosic polysaccharides was determined by HPAEC-PAD (high performance anion exchange chromatography–Pulsed Amperometric Detection) after acid hydrolysis of alcohol insoluble solid (AIS) (Figure 2b). As reported for other wheat tissues [40], monosaccharide composition of spike cell walls showed xylose as the main non-cellulosic constituent comprising 70–75 mol% of the total sugars, followed by arabinose (about 15%), glucose and galacturonic acid (about 5%), galactose (about 2.5%), and small contents of fucose, rhamnose and glucuronic acid (less than 1%). The comparison of the composition in monosaccharides between 02-5B-318_R_ and Saragolla_S_ spikes indicated a significantly higher percentage of arabinose, galactose and glucose as well as a lower percentage of xylose in the resistant line as compared to the susceptible one (Figure 2b). The arabinose/xylose ratio (Ara/Xyl), was significantly higher in spikes of 02-5B-318_R_ respect to Saragolla_S_ (Figure 2c). To identify the nature of cell wall polysaccharide differing in the two genotypes, AIS was sequentially fractionated by using solutions with increasingly harsh extraction conditions. Chelating Agent Soluble Solid (ChASS) fractions, mainly containing pectic polysaccharides, and 1 M KOH and 4 M KOH fractions, mainly containing hemicelluloses weakly and strongly bound to the cell wall, respectively, were isolated and analyzed for the monosaccharide composition (Table 2). Pectin fractions were not significantly different between the two genotypes. The hemicellulose-enriched fractions from the 02-5B-318_R_ plants contained a significantly higher amount of arabinose, galactose and glucose, a lower amount of xylose and showed a higher Ara/Xyl ratio in comparison with spikes from Saragolla_S_. In grasses, xylose and arabinose mainly constitute arabinoxylans (AX) and the combined levels of arabinose and xylose provide a good estimate of arabinoxylan content [16,41]. The percentage of arabinoxylans, calculated as sum of arabinose and xylose, was significantly lower in spikes of 02-5B-318R respect to Saragolla_S_ (Table 2). These results therefore indicate a significantly lower amount of arabinoxylans and higher degree of arbinoxylation in the hemicellulose of the 02-5B-318_R_ plants in comparison with Saragolla_S_ and that the differences previously observed between the two genotypes (Figure 2b) can be mainly attributed to the hemicellulose polymers. Monoclonal antibodies can be used to define structural features of polysaccharides in isolated cell wall fractions. In particular LM11 monoclonal antibody is specific to xylan domains enriched in arabinose substitutions [42]. 1 M KOH fractions extracted from spikes of 02-5B-318_R_ and Saragolla_S_ were analyzed with LM11 antibodies using immunodot assay. A higher level of LM11-binding epitopes was detected in 02-5B-318_R_ spikes in comparison with the FHB susceptible wheat genotype (Figure 2d) confirming the higher degree of xylan arabinosylation of 2-5B-318_R_ spikes in comparison with Saragolla_S_. A negative correlation between the Ara/Xyl ratio and wheat bran digestibility by fungal xylanases have been previously demonstrated [43]. In grasses, arabinose residues of xylans can form ferulic acid-mediated crosslinks between xylan chains and lignin components that limit the enzymatic digestibility of cell walls and improve *Fusarium* resistance [44-48]. The greater arabinosylation of xylans observed in 02-5B-318_R_ spikes could contribute to a lower degradability of these polymers during *Fusarium* infection and could consequently represent a potential cell wall trait contributing to FHB resistance. Recently, glycosyltransferases of family 61 were found to be arabinosyltransferases (XATs) in grasses [49]. Interestingly, arabinoxylan also influences desease resistance of barley against the powdery mildew fungus *Blumeria graminis* f. sp. *hordei* indicating that in monocot this hemicellulose is important in response to fungal infection [50]. The higher amount of glucose observed in 02-5B-318_R_ in comparison with Saragolla_S_ (Figure 2b and Table 2) indicate a different amount of (1,3;1,4)-β-D-glucan (Mixed linkage glucans; MLG) in their cell walls. Also in this case CslF and CslH glycosyltransferases implicated in MLG biosynthesis have been identified in grasses [51,52] Consistently, a decreased β-D-glucan content was observed in susceptible but not in resistant genotypes after inoculation of wheat spikes with *Fusarium culmorum* [53].

**Table 2:** **Monosaccharide composition of the ChASS, KOH 1 M and KOH 4 M fractions and Residues**

	**ChASS**		**KOH 1 M**		**KOH 4 M**		**Residue**	
	**R**	**S**	**R**	**S**	**R**	**S**	**R**	**S**
Fuc	1.4 ± 0.2	1.4 ± 0.2	nd	nd	nd	nd	nd	nd
Rha	4.5 ± 0.5	3.9 ± 0.4	0.11 ± 0.02	0.11 ± 0.01	0.26 ± 0.01	0.23 ± 0.03	0.38 ± 0.04	0.36 ± 0.07
Ara	22.8 ± 1.2	22.8 ± 1.9	14.4 ± 0.8	**11.3 ± 0.1**	12.9 ± 0.3	**11.4 ± 0.6**	16.5 ± 1.6	16.3 ± 0.6
Gal	21.1 ± 1.5	21.8 ± 0.8	2.2 ± 0.1	**1.7 ± 0.1**	1.9 ± 0.2	**1.2 ± 0.1**	3.1 ± 0.8	2.5 ± 0.3
Glu	7.9 ± 0.6	8.8 ± 0.9	10.4 ± 1.1	**6.6 ± 0.5**	9.5 ± 0.9	**5.9 ± 0.1**	11.7 ± 1.7	12.5 ± 2
Xyl	8.6 ± 0.5	8.7 ± 0.9	69.8 ± 1.2	**77.2 ± 0.6**	73.0 ± 1.5	**78.7 ± 0.7**	63.0 ± 2.2	62.6 ± 1.4
Man	10.2 ± 1.2	9.7 ± 0.9	nd	nd	nd	nd	nd	nd
GalA	21.9 ± 0.4	21.2 ± 0.7	2.8 ± 0.1	2.8 ± 0.2	2.4 ± 0.1	2.4 ± 0.1	5.1 ± 0.7	5.5 ± 1.3
GlcA	1.5 ± 0.1	1.5 ± 0.4	0.26 ± 0.02	0.27 ± 0.02	0.18 ± 0.02	0.18 ± 0.05	0.18 ± 0.01	0.21 ± 0.05
Ara + Xyl	---	---	83.2 ± 1.2	**86 ± 0.6**	84.9 ± 1.2	**89.1 ± 0.2**	75.6 ± 2.1	74.8 ± 2.6
Ara/Xyl	---	---	0.207 ± 0.014	**0.147 ± 0.002**	0.177 ± 0.007	**0.144 ± 0.009**	0.262 ± 0.032	0.259 ± 0.006

Monosaccharide composition of cell walls from spike of 02-5B-318_R_ and Saragolla_S_ wheat plants was determined by HPAEC-PAD. Values are expressed in mol% for each monosaccharide in each fraction. Value represent means ± SD (n = 4). Number in bold indicate statistically significant differences in each monosaccharides between the two genotypes, according to according to Student’s t-test (*p <* 0.05). ChASS, chelating agent-soluble solids; R = 02-5B-318_R_; S = Saragolla_S_.

### A different degree and pattern of methylesterification was observed in 02-5B-318_R_ and Saragolla_S_ spikes

**Figure 3:**
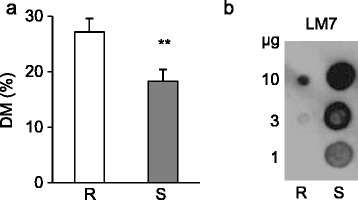
**Degree and pattern of pectin methylesterification (DM) in cell wall extracted from spikes of 02-5B-318**_**R**_ **and Saragolla**_**S**_ **plants. (a)** The DM was quantified and expressed as methanol to uronic acid molecular ratio (%). Data represent the average ± standard deviation (n = 6). Asterisks indicate data sets significantly different between 02-5B-318_R_ and Saragolla_S_ according to Student’s *t*-test (*p <* 0.01). **(b)** Immunodot analysis of pectin extracted from spikes of 02-5B-318_R_ and Saragolla_S_ plants using LM7 antibody. The micrograms of chelating agent soluble solid fractions from the two genotypes applied to the nitrocellulose membrane were indicated. The experiments were repeated three times with similar results. R = 02-5B-318_R_; S = Saragolla_S_.

The degree and pattern of pectin methylesterification impact the plant susceptibility to fungal and bacterial pathogens and affect the outcome of disease [20]. The degree of methylesterification (DM) of cell wall isolated from spikes of 02-5B-318_R_ was significantly higher (a about 30%) in comparison with Saragolla_S_ genotype (Figure 3a). In accordance with this, durum wheat plants overexpressing the pectin methylesterase inhibitor from kiwi, AcPMEI, exhibited a costitutive increased degree of methylesterification (DM) and were more resistant to *F. graminerum*, *Bipolaris sorokiniana* and *Claviceps purpurea* in comparison with untransformed plants [5,30]. It was also demonstrated that highly methylesterified pectins were less susceptible to the action of polygalacturonases (PGs) of both *B. sorokiniana* and *F. gram*inearum and a reduced growth of both fungal pathogens was detected on cell walls isolated from the transgenic plants indicating that the increased resistance of AcPMEI plants was due to the impaired ability of these fungi to colonize the host tissue [5]. Pectin domains with a random pattern of methylesterification, recognized by the monoclonal antibody LM7, have been demonstrated to be more sensitive to fungal PGs and pectate lyases (PLs) [54,55]. Immunodot assay performed with LM7 antibodies on ChASS enriched pectin fraction extracted from spikes of 02-5B-318_R_ and Saragolla_S_ showed a significant lower level of LM7-binding epitopes in the 02-5B-318_R_ plants in comparison with the susceptible genotype (Figure 3b). These results indicate that pectin of 02-5B-318_R_ spikes is enriched in domains less susceptible to PGs of *F. graminearum* secreted at early stages of infection [8]. Noteworthy, LM7 epitopes were also reduced in wheat plants overexpressing *AcPMEI* and showing improved resistance to *F. graminearum* [5].

**Figure 4:**
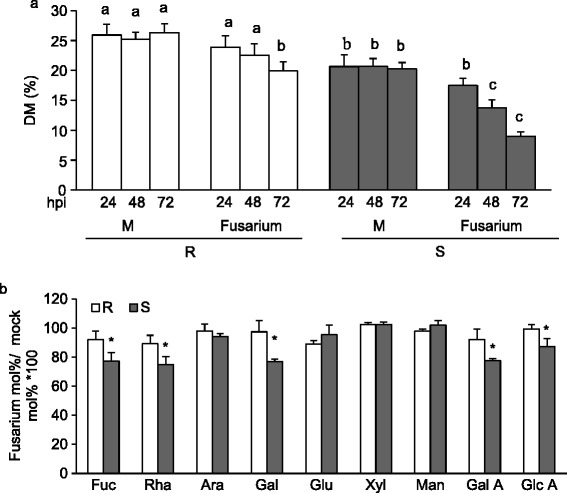
**Characterization of cell wall from spikes of 02-5B-318**_**R**_ **and Saragolla**_**S**_ **plants during** ***Fusarium*** **infection. (a)** Quantification of degree of pectin methylesterification (DM) at early stages of *Fusarium* infection. The DM was performed at the indicated hours post-inoculation (hpi). **(b)** Monosaccharide compositions of matricial polysaccharides was analysed at 72hpi; M, mock-inoculated plants; *Fusarium*, fusarium-inoculated plants. Data represent the average ± standard deviation (n = 6). The experiment was repeated twice with similar results. The different letters indicate datasets significantly different according to analysis of variance (ANOVA) followed by Tukey’s test (*p <* 0.05). Asterisks indicate data sets significantly different between 02-5B-318_R_ and Saragolla_S_ according to Student’s *t*-test (*p <* 0.05). R = 02-5B-318_R_; S = Saragolla_S_.

Recent evidence indicates that pectin de-methylesterification is induced at early stages of pathogen infection and favor the outcome of disease [56-58]. To determine whether pectin methylesterification is altered during fungal infection, DM was monitored at different times in uninfected and infected 02-5B-318_R_ and Saragolla_S_ spikes. The level of pectin methylesterification was significantly reduced in both genotypes during the early stages of *Fusarium* infection (Figure 4a). However, while a significant decrease of DM was observed in Saragolla_S_ spikes 48 h hour post inoculation (hpi), the DM reduction in 02-5B-318_R_ infected spikes was evident only after 72 hpi. Notably at 72 hpi, the reduction of DM in the susceptible Saragolla_S_ genotype was approximately 60% compared to a 25% reduction in the resistant genotype.

Studies focused on the analysis of modification of CW composition during fungal infection indicate that CW degradation occur in a sequential manner. Pectic enzymes, mainly including PGs and PLs, are the first to be produced by fungal pathogens during the early stages of infection followed by hemicellulases and cellulases [11,59,60] and although wheat contain a low level of pectin, PGs and PLs produced by *Fusarium* during infection are important determinants of the outcome of disease [8,61-63]. The analysis of the cell wall degradation by *F.graminearum* was performed by monitoring the monosaccharide composition of AIS isolated from infected spikes at different hpi (Figure 4b). No difference in monosaccharide composition was detected in the cell walls of infected spikes at 24 and 48 hpi (data not shown). At 72 hpi, the level of Fuc, Rha, Gal, GalA and GlcA monosaccharides was significantly reduced in Saragolla_S_ cell walls as compared to 02-5B-318_R_ indicating an higher extent of pectin degradation in the susceptible line. These results suggest that the higher DM and reduced content of pectin domains with random pattern methylesterification in 02-5b-318_R_ spikes as well as the reduced demethylesterification observed during infection can contribute to protect CW by fungal CWDEs degradation. The hemicellulose alteration was not observed at these stages of infection most likely, because the degradation of hemicelluloses occurs at late stages of infection as reported [8].

### Isolation and characterization of *WheatPME1*

The degree and pattern of pectin methylesterification *in planta* is regulated by PMEs. In addition to their important role in plant development [64,65] more recent evidence indicates that plant PMEs are directly involved in plant response against pathogens [56,57,66]. With the aim to identify wheat *PME* genes involved in *Fusarium* resistance, we focused our attention on *Brachypodium distachyon,* which is considered, in respect to vast majority of traits (i.e. cell wall composition, cell wall biosynthesis and plant-pathogen interactions), a convenient model system for monocots [67]. Among different PME sequences, identified using phytozome web site, we focused our attention on *Bradi1g16780.1* gene (hereafter named *BdPME1*). This gene showed the highest sequence similarity with wheat ESTs corresponding to a *PME* gene localized on the chromosome 2A, where the major FHB QTLs were found. The *BdPME1* complete genomic sequence consists of 1812 bp corresponding to a mRNA of 1728 bp encoding a 576 amino acids protein. BdPME1 belongs to type I PME containing, in addition to the catalytic PME domain, an N-terminal pro region that share homology with PMEIs [64,68]*. BdPME1* gene is located on chromosome 1 of *Brachypodium* genome and composed of two exons: the first at the 5’ end is 498 bp long including the pro region; the second including the PME domain is 1230 bp long. The two exons are separated by a very short intron sequence 84 bp long.

**Figure 5:**
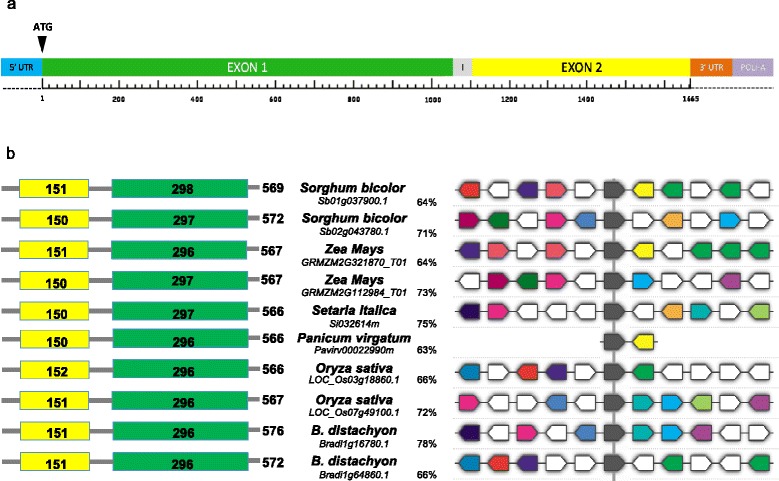
**Protein and gene structure of grasses PMEs. (a)** Schematic representation of *WheatPME1* structure in *Triticum aestivum* cv. Chinese Spring as predicted by FGENESH (http://linux1.softberry.com). In color the different gene regions. I = intron sequence of 84 bp; Exon 1 = 1053 bp; Exon 2 = 555 bp. **(b)** Protein and gene structures of grasses PME sequences related to *WheatPME1*. Left: graphic representation of PMEs; in yellow is indicated the pro region and in green the PME domain. Numbers inside the blocks indicate the lenght of aminoacid sequences. Right: Syntenic relationships among the *PME* genes; the black block indicates the most conserved nucleotide stretch showing the same position and orientation in all the grasses domain. For each PME, the plant origin, accession number and % of aa identity with respect to WheatPME1 are indicated.

With the aim to isolate the *BdPMEI1* orthologous in wheat, the gene sequence was blasted against public databases. Two wheat ESTs, showing a sequence identity higher than 80% with respect *BdPME1*, were found: the first one (BJ252439) entirely covered the *BdPME1* longer exon, while the second one (BJ246509) partially matched to the shorter exon at the 5’end of the gene sequence. The hexaploid wheat cv. Chinese Spring draft genome and the row 454 sequence reads of cv. Chinese Spring annotated at Cereals-DB archive (http://www.cerealsdb.uk.net) were searched to extend both ESTs and three larger consensus contigs were obtained assignable to each of the three A, B and D genomes. The three genes were identified using Softbarry prediction software (http://linux1.softberry.com) and named *WheatPME1-A, WheatPME1-B* and *WheatPME1-D* (Additional file 1: Figure S1). They showed a 99% nucleotide sequence identity among each other (Additional file 2: Figure S2) and the same intron/exons structure comprising two exons of 1053 and 555 bp, separated by an intron of 54 bp, corresponding to a mRNA sequence of 1608 bp (Figure 5a). The translation of the three *WheatPME1-A, WheatPME1-B* and *WheatPME1-D* sequences resulted in a same 537 amino acid protein, sharing an amino acid identity of 77% with BdPME1 (Additional file 1: Figure S1 and Additional file 3: Figure S3). The Propt.Comp. v.9.0 software indicates WheatPME1 as an “extracellular secreted protein”, conforming with the apoplastic locatization of the enzyme. The genomic sequences of *WheatPME1* homoeologous genes were obtained in 02-5B-318_R_ (A, B and D genomes) and Saragolla_S_ (A and B genomes) using genomic specific primers. The nucleotide sequences and intron/exons structures were respectively identical to the corresponding homoeologous *WheatPME1* genes in A, B and D genomes of 02-5B-318_R_ and in A and B genomes of Saragolla_S_ indicating that the sequence of this gene is strongly conserved in different wheat genotypes. No polymorphism in the *WheatPME1* gene was detected between 02-5B-318_R_ and Saragolla_S_. A BLAST search for plant sequences related to *WheatPME1* mRNA (BlastX, http://blast.ncbi.nlm.nih.gov) revealed a number of genes which predicted amino acid sequences were analyzed using non-redundant protein database. The search for grass sequences related to *WheatPME1* in Phytozome database (http://www.phytozome.net) revealed a number highly conserved PMEs genes, which encode proteins with a slightly variable length ranging from 566 aminoacids (in *Setaria italica, Panicum virgatum, Oryza sativa*) to 576 aminoacids (in *B. distachyon*) and with an identity level ranging from 63 to 78% (Figure 5b). All the selected PMEs belong to type I PME accounting for a smaller pro region at N-terminus of the *PME* gene, with length range of 151–153 aa, and a longer PME domain with length range of 297–299 aa; consistently with other evidence, these are highly conserved among the selected species [68]. Among the selected *WheatPME1* orthologous the gene structure appeared to be highly conserved (Figure 5b), and always composed by one single exon. The exceptions are rice and *Brachypodium distachyon* where the sequences are accounted for two gene copies, one is composed by one and another by two exons. Multi-alignment of genomic sequences showed that the different orthologous are characterized by several synthenic regions, particularly one of which showed the same position and orientation in all the selected grasses, likely corresponding to the active site of the enzyme (Figure 5b).

### *WheatPME1* gene chromosomal position and gene expression in 02-5B-318_R_ and Saragolla_S_ during *F.graminearum* infection

The chromosome position of the homoeologous *WheatPME1* genes was obtained using genetic stocks including nulli-tetrasomic, di-telosomic and a set of wheat deletion bin lines. The homeologous genes were physically located on the short arm of chromosome group 2 in 2BS1-0.53-0.75 and C-2AS5-0.78 bins, respectively. This chromosome position supports a role of *WheatPME1* gene in the control of *Fusarium* resistance since several major QTLs for FHB resistance have been found located in the same bin position with a R^2^ ranging from 3% to 27% [4].

**Figure 6:**
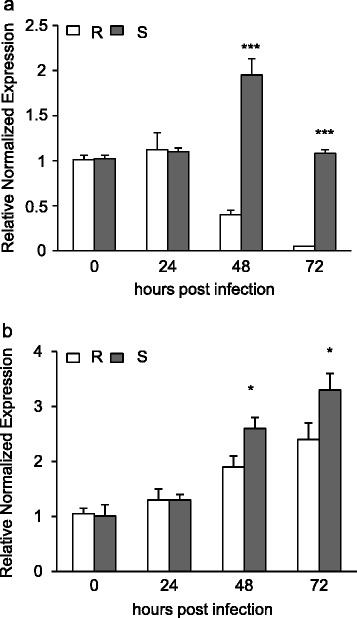
***WheatPME1*** **and** ***F. graminearum βTUB2*** **expression in spikes of resistant 02-5B-318**_**R**_ **and susceptible Saragoll**_**S**_ **wheat lines during infection. a)** *WheatPME1* expression was normalized to the average of four different internal references (*Actin*, *CDC*, *ADP-RF* and *RLI*) reported as fold-change with respect to the mock-inoculated control. **b)** *βTUB2* expression. The expression level was determined at 24, 48 and 72 hpi. Asterisks indicate data sets significantly different according to Student’s *t*-test (***p < 0.001; *p < 0.05). R = 02-5B-318_R_; S = Saragolla_S_.

To evaluate whether the expression of *WheatPME1* is modulated during *Fusarium* infection in 02-5B-318_R_ and Saragolla_S_, suitable primers were designed in a conserved region of the gene sequences in the three genomes and used for qRT-PCR analysis of transcripts from infected and mock-inoculated spikes. *WheatPME1* expression level was measured at 0, 24, 48 and 72 hours post inoculation (hpi). In both wheat lines, the *WheatPME1* expression level at 24 hpi did not show significant difference in comparison with the mock-inoculated controls (Figure 6). In 02-5B-318_R,_ the level of *WheatPME1* expression tends to decrease showing a 1-fold lower expression at 72 hpi. It is possible that during Fusarium infection, plants down regulate *WheatPME1* to ensure a higher degree of CW methylesterification which would protect the CW against *Fusarium* pectic enzymes. On the contrary, in susceptible Saragolla_S_ spikes the expression level of *WheatPME1* showed a 2-fold increase at 48hpi in comparison with the non-infected control, and then dropped back to the basal expression level. Consistently with this observation, the analysis of Wheat 61 k GeneChip annotated at PLEXdb database (http://www.plexdb.org) indicated that the expression of *WheatPME1* is only induced by *Fusarium* in the susceptible hexaploid wheat cv. Chinese spring but not in a line carrying a resistance locus from the wild *Thinopyrum elongatum* chromosome 7E [69] which supports the involvement of this specific PME isoform in wheat response to FHB. The induced expression of *WheatPME1* in the susceptible Saragolla_S_ line at 48 hpi likely contributes to the observed greater reduction of pectin methylesterification and increased pectin degradation in comparison with 02-5B-318_R_, making Saragolla_S_ CWs likely more susceptible to fungal CWDEs action and tissue more accessible to fungal colonization. *Fusarium* growth was assessed by measuring the expression of *beta-tubulin 2* gene (βTUB2; FJ526863.1) in spikes from infected and mock-inoculated 02-5B-318_R_ and Saragolla_S_ plants (Figure 6). The βTUB2 expression showed increased levels at 24, 48 and 72 hpi in both inoculated lines, however, to a higher extent in the susceptible Saragolla_S_ reflecting an increased fungal growth in these plants. This result also indicates that the repression of *WheatPME1* observed in 02-5B-318_R_ was most likely, due to a negative regulation of the gene.

## Conclusions

Different mechanisms of disease resistance of wheat against *F. graminearum* have been elucidated, mainly in common wheat. These include the specific activation of defense signaling pathways, detoxification/ tolerance and resistance to fungal toxins, and the induction of plant defense secondary metabolites [70,71]. Durum wheat is one of the most susceptible cereals to *F.graminearum* infection and breeding for the FHB resistance is complicated by the lack of resistance sources. It was speculated that durum wheat either lacks resistance genes or carries effective susceptibility factors and/or suppressor genes that compromise FHB resistance [72,73].

Emerging evidence indicates that content and composition of cell wall polymers affect the susceptibility of cell wall to CWDEs and can play a role in the outcome of host-pathogen interactions [11-14]. In this study we provide a comprehensive overview of cell wall composition of spikes at anthesis, a key developmental stage particularly susceptible to *Fusarium* infection, from a resistant common wheat and a susceptible durum wheat genotypes. The comparative CW analysis revealed constitutive differences in monolignol composition of lignin, with a higher amount of S-type lignin present in the resistant 02-5B-318_R_ wheat as compared to the Saragolla_S_ susceptible plants. We also detected differences in hemicellulose and pectic polymers of the cell wall in spikes of the two genotypes. In particular, resistant line was enriched in AXs with a higher degree of arabinose substitution. The CW of resistant line contained a higher amount of methylesterified pectin with a less random distribution of methylated GalA.

The analysis of degree of methylesterification and monosaccharide composition of the cell wall of spikes at early stages of *Fusarium* infection indicated an higher demethylesterification and an higher extent of pectin degradation in the susceptible line as compared to 02-5B-318_R_. We propose that cell wall differences between the susceptible and resistant genotype could contribute to the different polysaccharide degradation we observed at early stage of *F.graminearum* infection as well as could influence the outcome of the disease. Cell wall genes which regulating the cell wall traits identified could be involved in FHB resistance. Among these genes, *WheatPME1* was identified, characterized and proposed to participate in the control of pectin methylesterification during the interaction of wheat with *F. graminearum.* In addition to the cell wall components here identified, other cell wall traits are known to be involved in monocot resistance to Fusarium [70]. Examples are the cell wall‐bound thionins, having growth inhibition activity toward pathogens as well as callose and structural hydroxyproline‐rich glycoproteins, both involved in cell wall reinforcement at the site of pathogen infection [14,74,75]. Moreover, inhibitors of CWDEs such as polygalacturonase inhibiting proteins (PGIPs), PMEIs, *Triticum aestivum* xylanase inhibitors (TAXIs) and xylanase inhibitor proteins (XIPs), influencing cell wall degradability during infection, have been associated to wheat resistance against Fusarium [6,11,76]. All these cell wall traits are potential molecular markers useful in plant breeding programs targeted to the selection of wheat varieties with a durable resistance to Fusariosis.

## Methods

### Growing condition of wheat and pathogenicity tests

Wheat seeds were surface-sterilized in Sodium hypochlorite and transferred on petri dishes containing 3MM paper soaked with water. Plates were stored at 4°C in the dark for 24-48 h and transferred in a growth chamber at 23°C in the dark for 15 days. Plants grown in a controlled environmental chamber maintained at 22°C, 70% humidity with a 16 hours photoperiod (300μE m-2 s-1).

Pathogenicity tests were conducted using the *Fusarium* resistant common wheat line, accession n. 02-5B-318 (a breeding line derived from cv. Sumai3, kindly provided by dott. Stefano Ravaglia, S.I.S., Bologna, Italy) and on the susceptible durum wheat cv. Saragolla. Uniform inoculum pressure was applied during flowering by using the *Fusarium graminearum* PH 1 isolate (kindly provided by prof. Quirico Migheli, University of Study of Sassari, Italy). Plants were artificially inoculated by spraying on each plants 100 mL of a suspension containing a mixture of conidia of *F. graminearum* (about 1.0 × 105 conidia per mL). *Fusarium* strain was grown for one week on PDA (Potato Dextrose Agar) and conidia were isolated by growing pieces of mycelium in shaking cultures in 2 L PIREX flasks containing 1 L sterile CMC (Carboxyl-methyl-cellulose) medium (15gr CMC, 1gr NH_4_NO_3_; 1gr KH_2_PO_4_; 0.5 gr MgSO_4_*7H_2_0; 1gr yeast extract; 50 ug/mL chloramphenicol). After 5-day incubation in the dark at 25°C shaking at 150 rpm, flasks content was filtered through two layers of cheesecloth by centrifugation at 3,000 rpm for 10 min; pellet was re-suspended in sterile water and centrifuged again. Filtered conidia were finally re-suspended in 10 mL of sterile water. The concentration of the inoculum was measured with a Burker camera (HBG Henneberg-Sander GmbH, Lutzellinden, Germany) using a light-microscope.

Twenty plants for 02-5B-318 and Saragolla line were artificially spray-inoculated during anthesis with a 10^6^/mL distilled-water macroconidia suspension, for each plant 5 spikes were chosen for a total of 100 spikes per lines. *Fusarium* head blight (FHB) incidence and severity were recorded five, ten and twenty days after inoculation on both infected and mock-inoculated (controls) wheat plants: FHB severity was averaged as the percentage of infected spikelets per plant, while FHB incidence was averaged as the number of infected spikes per plant; a mean value of at least 20 plants per genotype was assessed. Infection experiments were statistically evaluated by performing analysis of variance followed by the Student’s *t* test.

### Alcohol-insoluble solids (AIS) extraction

Wheat spikes were collected at anthesis stage and infected spikes were collected after 24, 48 and 72 hours post Fusarium inoculation. Tissues excised from the central part of each spike, including rachis and spikelets were ground to a fine powder with a mortar and pestle in presence of liquid nitrogen. Milled tissue (200 mg) was washed twice in a pre-warmed (70°C) 70% ethanol, vortexed, and pelleted by centrifugation at 25,000 g for 10 min. The pellet was suspended with a chloroform:methanol mixture (1:1, v/v) and shaked for 30 min at room temperature. Samples were pelleted by centrifugation at 25,000 g for 10 min. Pellets were re-suspended in 1 ml 80% acetone and spin at 25,000 g for 5 min. Supernatants were discarded and pellets were dried at room temperature over-night.. Starch was removed by treating the AIS with the porcine Type I-A α-amylase (100 U g-1 AIS; product number A4268; Sigma-Aldrich) in a 100 mM potassium phosphate buffer pH 7.5 mM NaCl and 0.02% (w/v) NaN3 for 24 hours at 37°C. The suspension was centrifuged at 25,000 × g for 20 minutes, and pellet was then washed with distilled water and 80% acetone.

### Lignin content and monolignol composition

Acetyl bromide lignin in de-strached AIS from the spikes of both wheat varieties was determined according to [77] with some modifications. Briefly, 3 mg of AIS were placed in glass vials, and then 200 μl 25% acetyl bromide in acetic acid and 600 μl of acetic acid (glacial) were added. Mixtures were incubated at 50°C for 2 h, with occasional shaking. 15 μl of reaction mixture after cooling was transferred to 96-well plate (UV transparent), and 15 μl 0.3 M NaOH, 5 μl 0.5 M hydroxylamine hydrochloride and 65 μl acetic acid (glacial) were added. After shaking, optical density at 280 nm against blanks (all reagents without AIS samples) was measured using plate reader. Lignin concentration was determined using the following equation: % lignin content = (absorbance × 100)/SAC × AIS concentration (g^−1^) where SAC is the specific absorption coefficient of lignin [78]. Specific monolignol composition was determined using Pyrolysis-GC-MS. De-starched AIS (3 mg) were single-shot pyrolized at 500°C and the volatile compounds were separated on HP-5 MS column (30 m × 0.25 mm, Agilent Technologies Inc, USA) using GC system (6890 N GC-system interfaced to 5975B inert MSD, Agilent Tech., USA). Oven temperature was initially set at 50°C and ramped to 280°C over a period of 53 min. Helium was the carrier gas for the volatile compounds and the split ratio was set at 50:1. Peak identification was performed by comparison of sample spectra with those published by [79]. The monolignol composition was calculated as %, combining the peak areas of similar type of lignin.

### Determination of the degree of methylesterification

De-starched AIS (4 mg) were saponified by suspending them in 60 μl H_2_O up and 20 μl of 1 M NaOH. The solution was incubated at room temperature for 1 h and afterward neutralized with HCl. After centrifugation at 25,000 × *g*, aliquots of the supernatant (50 μl) of 02-5B-318_R_ and Saragolla_S_ were loaded in microtiter plates (96-well cod.9018 from Costar, Cambridge, MA, U.S.A.). Alcohol oxidase (50 μl) was added to each well (0.03 units in 0.1 M sodium phosphate, pH 7.5) (Sigma, St. Louis), and this mixture was incubated at room temperature for 15 min on a shaker. Thereafter, 100 μl of a mixture containing 0.02 M 2,4-pentanedione in 2 M ammonium acetate and 0.05 M acetic acid was added to each well. After 10 min of incubation at 68°C, samples were cooled on ice and absorbance was measured at 412 nm in a microplate reader (ETI-System reader; Sorin Biomedica Cardio S.p.A., Saluggia, Italy. The amount of methanol was estimated as described [80]. For uronic acid quantification, 4 mg saponified AIS samples were incubated in 200 μl of 2 M Trifluoracetic acid (TFA) at 121°C. After 1.5 hours, 200 μl of isopropanol was added and the mixtures evaporated at 40°C with a stream of N_2_ gas. This step was repeated twice and samples were dried at room temperature overnight. The TFA hydrolyzed monosaccharides were suspended in 200 μl of water and the Uronic acid content in the supernatant was quantified colorimetrically using the automated sulfamate/m-hydroxy diphenyl assay [81] and galacturonic acid (Fluka 48280) as standard. The degree of methylesterification was expressed as methanol to uronic acid molar ratio (%).

### Cell wall fractionation and monosaccharides composition

To isolate fractions enriched in various cell wall components, AIS were subjected to sequential extraction buffers (at final concentration of 30 mg/ml) in constant mixing for 24 hours at room temperature. The following order was followed: 50 mM ammonium oxalate (Chelating Agent Soluble Solid, ChASS) pH 5.2 with 0.02% sodium azide; 1 M KOH, 1% (w/v) of sodium borohydride with 0.02% sodium azide and 4 M KOH with 1% (w/v) of sodium borohydride with 0.02% sodium azide. The 1MKOH and 4 M KOH fractions were neutralized using glacial acetic acid. All of the extracts were dialyzed against four changes of 4 L of deionized water and then lyophilized. For each genotype six independent replicates were analyzed. The monosaccharide composition of destarched AIS, the ChASS, 1 M KOH, 4 M KOH fractions and of residue, all hydrolysed with TFA was determined by HPAEC-PAD using a PA20 column (Dionex, CA, USA). Peaks were identified and quantified by comparison to a standard mixture of rhamnose (Rha), arabinose (Ara), fucose (Fuc), galactose (Gal), glucose (Glc), xylose (Xyl), mannose (Man), galacturonic Acid (GalUA),and glucuronic acid (GlcUA) (Sigma-Aldrich).

The crystalline cellulose was determined as previously described [82]. The cellulose derived glucose content in destarched AIS was determined by an anthrone colorimetric assay [83] with glucose (Sigma G8270) as a standard.

### Immunodot assay

For each experiment, ChASS and KOH 1 M fractions were applied as 1 μL aliquots to nitrocellulose membrane (0.45 μm pore size; Bio-Rad, Hercules, CA, USA) in a threefold dilution series. Arrays were incubated for 1 hour in 5% (w/v) milk protein (MP; Bio-Rad) in PBS pH 7.8 (MP-PBS), and probed for 1.5 hours with primary LM7 and LM11 monoclonal antibodies (purchased from PlantProbes, Paul Knox Cell Wall Lab, University of Leeds,Leeds, UK) diluted 1:20 in 3% MP-PBS. After extensive washes in PBS, arrays were incubated with anti-rat conjugated to horseradish peroxidase (A7058; Sigma-Aldrich) diluted 1:1000 in MP-PBS buffer. After washing in PBS, LM11 arrays was developed using 4-chloro-1-naphthol [84] and, due to a weak signal, LM7 was developed using ECL detection reagent (Amersham).

### Bioinformatic analysis

In order to identify homologous proteins to wheat methylesterase enzyme, a bioinformatic analysis was carried out on grass species (*Sorghum bicolor*, *Zea mays*, *Setaria italica*, *Panicum virgatum*, *Oryza sativa* and *Brachypodium distachyon*) annotated in Phytozome v.9.1 database (http://www.phytozome.net). *Brachypodium BdPME1* complete genomic sequence was used as the initial query in a BLAST-search against wheat EST (Expressed Sequence Tags) database at NCBI (http://blast.ncbi.nlm.nih.gov), with the aim to retrieve sequences with a high similarity score (>80%). Each suitable EST was finally searched for similarity in the Chinese Spring database at Cereal DB (http://www.cerealsdb.uk.net/search_reads.htm), to extract 454 reads and obtain larger consensus contigs of the hexaploid reference cultivar using an e-value cut-off of e^−5^.

### Isolation and characterization of *WheatPME1* sequence in wheat lines

*WheatPME1* gene isolation was conducted in the 02-5B-318 accession of *T. aestivum* and in the durum wheat cv. Saragolla, respectively FHB-resistant and susceptible. Genomic DNA was isolated from the two wheat lines according to the extraction protocol by [85] starting from 0.1 gr of fresh leaves, then checked for quality and concentration at a Nanodrop device (Thermo Scientific, Walthman, MA, USA). Purity of extracted DNA was assessed by measuring 260 nm/280 nm ratio, with a value of approximately 1.8-2 indicating a good quality.

Genomic DNA was PCR-amplified with several primer pairs opportunely designed by OligoExplorer software on *Brachypodium* genomic sequence, Chinese Spring ESTs and consensus contigs, in order to cover the entire gene sequence. All the amplification reactions were initially carried out in a gradient of annealing temperature in order to check for primer specificity and identify the optimal annealing conditions for each primer combination. PCR reactions were conducted in a total volume of 25 μl containing 100 ng of template gDNA, 250 nmol/L of each primer, 1X reaction Buffer (10 mmol/L Tris–HCl, pH 8.3; 10 mmol/L KCl), 200 μmol/L of each dNTP, 2.5 mmol/L of MgCl_2_, and 1 unit of *Taq* DNA polimerase (*EuroTaq*, Euroclone®). Amplifications were run in a *MyCycler™ Personal Thermal Cycler* (Bio-Rad®) according to the following protocol: 5 min at 95°C, followed by 32 cycles of: 1 min at 95°C, 1 min at the given annealing temperature, and 2 min at 72°C, followed by a final extension step of 15 min at 72°C. Finally, PCR products were checked for the expected molecular size by visualization on 1.5-2% agarose gel stained with Gel-Red® dying solution (Biotium, Inc., Hayward, CA).

For the chromosomal localization of *WheatPME1* genes, nulli-tetrasomic lines (NTs) of *Triticum aestivum* cv. Chinese Spring [86,87] were used to physically localize *PME* markers to chromosomes. Chinese Spring di-telosomic lines [88] were used for the assignment of markers to each chromosomal arm. Physical location on chromosome bins of each PCR fragment was obtained using a set of common wheat deletions lines dividing genome chromosomes into bins (kindly provided by B. S. Gill, USDA-ARS, Kansas State University) [89]. Single-band PCR products were directly purified from a volume of about 100 μl using the *EuroGold Cycle Pure Kit* (Euroclone®) following the manufacturer instructions, with the only exception of using sterile deionized water rather than the supplied elution buffer, to increase the efficiency of following sequencing reactions. Purified DNA fragments were checked on 1.5-2% agarose gel stained with Gel-Red® dye solution, then evaluated for concentration by detecting absorbance at a 260 nm wave length at a Nano Drop device (Thermo Scientific®). Sequencing analyses were performed for each fragment in both strands by BMR Genomics S.r.l (Padova). Sequence assembly was obtained with *Codone Code Aligner* and *Geneious* softwares. Multi-alignments of gene sequences between 02-5B-318 and Saragolla were carried out by *ClustalW* (http://www.ebi.ac.uk) and BLAST (http://blast.ncbi.nlm.nih.gov). Gene structure prediction was performed by the FGENESH on-line tool (http://linux1.softberry.com/cgi-bin/programs/gfind/bestorf.pl).

### Gene expression analysis

Total RNA was isolated from spikes of infected and mock-inoculated (control) plants of both resistant 02-5B-318 and susceptible Saragolla at 24, 48 and 72 hours post inoculation. For each sample three biological replicates were collected from different plants. Tissues were harvested in each phase, immediately frozen in liquid nitrogen and stored at −80°C until RNA extraction. Total RNA was extracted using the *RNeasy Plant Mini Kit* (Qiagen®) and checked on 1.5% denaturing agarose gel; amount and purity were determined with a Nano-Drop spectrophotometer. All RNA samples were led to the same concentration (1 μg/μl) and reverse-transcribed into double stranded cDNA by using the *Quanti-Tect Reverse Transcription Kit* (Qiagen®) following the manufacturer instructions, after a prior treatment with a *DNA Wipeout Buffer* for the removal of gDNA contamination.

Primer pairs were designed by using OligoExplorer software on a conserved *pme* nucleotide region between the three wheat genomes, in order to determine the total pectin methyl-esterase gene expression in the two wheat lines. As shorter amplicons work more efficiently, primers were designed to amplify small DNA fragments in the range of 50–200 bp. *Actin*, *CDC* (Cell Division Control), *ADP-RF* (ADP-Ribosilation Factor) and *RLI* (RNase L Inhibitor-like protein) genes were used as internal references to normalize *PME* expression data. Specific primers for *Fusarium β-tubulin 2* (*βTUB2*) gene were used to assay fungal infection in both inoculated and non-inoculated wheat samples (Additional file 4: Table S1).

In order to identify the best temperature to ensure primer specificity, standard PCR on cDNA were performed with a gradient of annealing temperatures (ranging between 55°C and 65°C) for both target and reference primer pairs, by using high fidelity *MyTaq DNA polymerase* (BioLine). Amplicon specificity was confirmed for each primer pair by checking the presence of single PCR products of expected molecular size on 2% (w/v) agarose gel stained with Gel Red® dying solution, and by direct sequencing of the amplified fragments (BMR Genomics, Padova, Italy).

Primer concentration was optimized for each gene in preliminary Real-Time amplification experiments by running reactions with different combinations of forward and reverse primers in the final mix (100, 300, 500 and 900 nM), then choosing those giving the highest endpoint fluorescence and a low Cq value. Primer specificity was also checked by performing melting curves of PCR products following Real Time amplifications.

qRT-PCR reactions were performed using EvaGreen® chemistry in the CFX96™ Real-time PCR System (Bio-Rad) following these conditions: 95°C for 3 min, followed by 40 cycles of: 95°C for 10 sec and 60°C for 30 sec. In each qPCR experiment 1 μl of a 1:10 dilution of cDNA was used in a final volume of 10 μl containing 5 μl of SsoFast EvaGreen® SuperMix 10X (Bio-Rad) and a primer concentration of 500 nM for *WheatPME1*, and 100 nM for *Actin*, *CDC*, *ADP-RF* and *RLI*. Three independent amplification reactions (technical replicates) were carried out for each biological replicate.

PCR reaction efficiency was calculated for both target and reference genes by generating six-point standard curves of three-fold serial dilutions of cDNA. Standards were run in the same amplification plate of the unknown samples. All experiments were performed in Hard-Shell 96-well skirted PCR plates (HSP9601) with Microseal® ‘B’ Adhesive Seals (MSB-1001) from Bio-Rad®.

Data analyses were performed with the CFX Manager™ 3.1 software, using the Normalized Expression mode (ΔΔC_q_) which calculated the relative quantity of target (*WheatPME1*) normalized to the relative quantity of internal references (geometric mean of multiple reference genes). For both target and reference genes, relative expression was calculated as fold-change respect to the mock-inoculated controls at each harvesting stage, and determining the standard deviation (SD) for the relative quantity. All the results were analyzed by ANOVA.

### Availability of supporting data

All the supporting data are included as additional files in this manuscript.

## Additional files

Additional file 1: Figure S1.
*WheatPME1*genes and protein sequences. Fasta sequences of hexaploid cv Chinese Spring *WheatPME1-A*, *WheatPME1-*B and *WheatPME1-D* genes and encoded polypeptides. Additional file 2: Figure S2. Multiple alignment of *WheatPME1* genes identified in *Triticum aestivum*, cv. Chinese Spring in the corresponding A, B and D genomes. In yellow are highlighted the SNPs between the A/D and B genomes. Additional file 3: Figure S3. Multiple alignment of *WheatPME1* from A, B and D genomes of *Triticum aestivum* cv. Chinese Spring and from *Brachypodium distachyon* (*Bd*PME1). The yellow box indicates the pro region, whereas the green box corresponds to the PME domain. The protein is reported in C terminus-N terminus orientation. Additional file 4: Table S1. Primer pairs sequences for housekeeping and target genes.

## Abbreviations

FHB: Fusarium Head Blight
CW: Cell wall
CWDEs: Cell wall degrading Enzymes
PME: Pectin Methylesterase
PMEI: Pectin Methylesterase inhibitor
XIP: Xylanase inhibitor protein
PGIP: Polygalacturonase inhibiting protein
TAXI: *Triticum aestivum* xylanase inhibitor
QTL: Quantitative trait Loci
EST: Expressed sequence tags
CDC: Cell division control
ADP-RF: ADP-ribosilation factor
RLI: RNase L inhibitor-like protein
βTUB2: β-tubulin 2
SD: Standard deviation
Cq: Quantification cycle
qRT-PCR: Quantitative reverse-transcription PCR
