# Supplementary material for: Cell wall traits as potential resources to improve resistance of durum wheat against Fusarium graminearum
Source: BMC Plant Biol. 2015 Jan 19;15:6. doi: 10.1186/s12870-014-0369-1 (PMC4298115; doi:10.1186/s12870-014-0369-1)
Supplement: Additional file 1: Figure S1. — WheatPME1genes and protein sequences. Fasta sequences of hexaploid cv Chinese Spring WheatPME1-A, WheatPME1-B and WheatPME1-D genes and encoded polypeptides. [file 12870_2014_369_MOESM1_ESM.pdf]

>*Triticum aestivum* cultivar Chinese *WheatPME1-A* gene

ATGAGTAAAGGTGCGATCATCGGCGCGTCGACCGTCCTGGTGGTGGCGGT
CGTCGCCGCCGTGTGCGTCGTGTCCTTCAAGGGCAACGGCGGCGACAAGG
GCGATGGGGAGCTCACCACCTCCGTCAAGTCCGTCAAGGCCTTCTGCCAG
CCCATGGACTACAAGGAGACGTGCGAGGCGGAGCTGACCAAGGTGGGCGG
CAACGCCACGTCGCCGACGGAGCTCGCCAAGGCCATCTTCGAGGTGACCT
CGGAGAAGATCAGGAAGGCCATCAGCGAGTCGGCCACCCTGGAGGAGCTC
AAGAGCGACCCGCGCACGTCGGGGGCCCTCGAGAACTGCAAGGAGCTGCT
GGAGTACGCCATCGAGGACCTCAAGACCACCTTCGACCGCCTCGGCGGCT
TCGAGATGACCGACTTCAACAAGGCCGCCGCCGACCTCAAGACCTGGCTC
AGCGCCGCGCTCACCTACCAGGAGACCTGCCTCGACGGCTTCGCCAACAC
CACCACCGACGCCGCCGCCAAGATGCGCGGCGCGCTCAACGCCTCCCAGG
AGCTCACCGAGGACATCCTCGCCGTGGTCGACCAGTTCTCCGCCTCCCTC
GGCAGCCTCAACATCGGGAAGAGGAGGCTGCTAGGGGAGGAGGACGGCAT
GCCTTACTGGATGAACGACGGGAAGAGGCGGCTGCTGGAGGCCGAGCCCT
CCGCACCCGAGTTCAAGCCCAACGTCACCGTCGCCGCTGACGGCAGCGGC
GACTTCAAGACCATCAAGGAGGCGCTCGCAAAGGTGCCGCCCAAGAGCGC
CTCCATGTACGTCATGTACATCAAGGCCGGCACCTACAAGGAGTACGTCT
CCGTGGGCCGCCCTATCACCAACCTCGTCGTCATCGGCGACGGCGACGAC
AAGACCATCATCACCGGCAACAAGAACTTCAAGATGAACATCACCACCAA
GGACACCGCAACCATGGGAGGCAATCGGGAACGGCTTCTTCATGAAGGGC
GTGAGGGTGGAGAACACGGCGGGGGCCGAGAACCACCAGGCCGTGGCACT
GCGGGTGCAGAGCGACCAGGCCGTCTTCTACCAGTGCTACTTCGACGGGT
ACCAAGACACGCTCTACACCCACGCGCAACGGCAGTTCTTCCGCGACTGC
ACCGTCACCGGCACCATCGACTTCATCTTCGGCAACTCCCAGGTGGTCAT
CCAGAACTGCCTCATCCTGCCGCGCAAGCCCATGGACAACCAGCTCAACA
TCATCACCGCGCAAGGGCGGCGCGAGAAGCGCTCCGTCGGGGGCACCGTC
ATGCACAACAACACCATCGAGCCGCACCCGGACTTCAAGGACTCGACGGG
CAAGATCAAGACCTACCTGGCGCGGCCATGGAAGGAGTACTCTAGGACCA
TCTACATCCAGAACGAGATCGGCGCCTTCATCGACCCCAAGGGCTGGCTC
GAGTGGAACGGCGACTTCGCCCTCGAGACCCTCTTCTACGCCGAGGTGGA
GAACACCGGGCCCGGCGCCGATATGAGCCAGCGCGCCAAGTGGGGCGGCA
TCAAGACTGTCACCTACGCTGACGCACAAAAGGAGTACACCGTCGAGGCC
TTCATCCAGGGCGAGCAGTTCATCCCCAAGTACGGCGTGCCCTACATCCC
GGGGCTCCTCCCGCAGTCGGAGGCGGGGAGGG

>*Triticum aestivum* cultivar Chinese *WheatPME1-A* predicted protein

MSKGAIIGASTVLVVAVVAAVCVVSFKGNGGDKGDGELTTSVKSVKAFCQ
PMDYKETCEAELTKVGGNATSPTELAKAIFEVTSEKIRKAISESATLEEL
KSDPRTSGALENCKELLEYAIEDLKTTFDRLGGFEMTDFNKAAADLKTWL
SAALTYQETCLDGFANTTTDAAAKMRGALNASQELTEDILAVVDQFSASL
GSLNIGKRRLLGEEDGMPYWMNDGKRRLLEAEPSAPEFKPNVTVAADGSG
DFKTIKEALAKVPPKSASMYVMYIKAGTYKEYVSVGRPITNLVVIGDGDD
KTIITGNKNFKMNITTKDTATMGGNRERLLHEGREGGEHGGGREPPGRGT
ADTLYTHAQRQFFRDCTVTGTIDFIFGNSQVVIQNCLILPRKPMDNQLNI
ITAQGRREKRSVGGTVMHNNTIEPHPDFKDSTGKIKTYLARPWKEYSRTI
YIQNEIGAFIDPKGWLEWNGDFALETLFYAEVENTGPGADMSQRAKWGGI
KTVTYADAQKEYTVEAFIQGEQFIPKYGVPYIPGLLP

> *Triticum aestivum* cultivar Chinese *WheatPME1-B* gene

ATGAGCAAAGGCGCCATAATCGGCGCGTCGACCGTCCTGGTGGTGGCGGT
CGTCGCCGCCGTCTGCGTCGTGTCCTTCAAGGGCAACGGCGGCGACAAGG
GCGATGGGGAGCTCACCACCTCCGTCAAGTCCGTCAAGGCCTTCTGCCAG
CCCATGGACTACAAGGAGACGTGCGAGGCGGAGCTGACCAAGGTGGGCGG
CAACGCCACGTCGCCGACGGAGCTCGCCAAGGCCATCTTCGAGGTCACCT
CGGAGAAGATCAGGAAGGCCATCAGCGAGTCGGCCACCCTGGAGGAGCTC
AAGAGCGACCCGCGCACGTCGGGGGCCCTCGAGAACTGCAAGGAGCTGCT
GGAGTACGCCATCGAGGACCTCAAGACCACCTTCGACCGCCTCGGCGGCT
TCGAGATGACCGACTTCAACAAGGCCGCCGCCGACCTCAAGACCTGGCTC
AGCGCCGCGCTCACCTACCAGGAGACCTGCCTCGACGGCTTCGCCAACAC
CACCACCGACGCCGCCGCCAAGATGCGCGGCGCGCTCAACGCCTCCCAGG
AGCTCACCGAGGACATCCTCGCCGTGGTCGACCAGTTCTCCGCCTCCCTC
GGCAGCCTCAACATCGGGAAGAGGAGGCTGCTAGGGGAGGAGGACGGCAT
GCCTTACTGGATGAACGACGGGAAGAGGCGGCTGCTGGAGGCCGAGCCCT
CCGCACCGGAGTTCAAGCCCAACGTCACCGTCGCCGCTGACGGCAGCGGC
GACTTCAAGACCATCAAGGAGGCGCTCGCAAAGGTGCCGCCCAAGAGCGC
CTCCATGTACGTCATGTACATCAAGGCCGGCACCTACAAGGAGTACGTCT
CCGTGGGCCGCCCTATCACCAACCTCGTCGTCATCGGCGACGGCGACGAC
AAGACCATCATCACCGGCAACAAGAACTTCAAGATGAACATCACCACCAA
AGACACCGCAACCATGGGAGGCGATCGGGAACGGCTTCTTCATGAAGGGC
GTGAGGGTGGAGAACACGGCGGGGGCCGAGAACCACCAGGCCGTGGCGCT
GCGGGTGCAGAGCGACCAGGCCGTCTTCTACCAGTGCTACTTCGACGGGT
ACCAAGACACGCTCTACACCCACGCGCAACGGCAGTTCTTCCGCGACTGC
ACCATCACCGGCACCATCGACTTCATCTTCGGCAACTCCCAGGTGGTCAT
CCAGAACTGCCTCATCCTGCCGCGCAAGCCCATGGACAACCAGCTCAACA
TCATCACCGCGCAAGGGCGCCGCGAGAAGCGCTCCGTCGGGGGCACCGTC
ATGCACAACAACACCATCGAGCCGCACCCCGACTTCAAGGACTCGACGGG
CAAGATCAAGACCTACCTGGCGCGCCCATGGAAGGAGTACTCCAGGACCA
TCTACATCCAGAACGAGATCGGCGCCTTCATCGACCCCAAGGGCTGGCTC
GAGTGGAACGGCGACTTCGCCCTCGAGACCCTCTTCTACGCCGAGGTGGA
GAACACCGGGCCCGGCGCCGACATGAGCCAGCGCGCCAAGTGGGGCGGCA
TCAAGACTGTCACCTACGCCGACGCGCAGAAGGAGTACACCGTCGAGGCC
TTCATCCAGGGCGAGCAGTTCATCCCCAAGTACGGCGTGCCCTTCATCCC
GGGGCTCCTCCCGCAGTCGGAGGCGGGGAGGG

>*Triticum aestivum* cultivar Chinese *WheatPME1-B* predicted protein

MSKGAIIGASTVLVVAVVAAVCVVSFKGNGGDKGDGELTTSVKSVKAFCQ
PMDYKETCEAELTKVGGNATSPTELAKAIFEVTSEKIRKAISESATLEEL
KSDPRTSGALENCKELLEYAIEDLKTTFDRLGGFEMTDFNKAAADLKTWL
SAALTYQETCLDGFANTTTDAAAKMRGALNASQELTEDILAVVDQFSASL
GSLNIGKRRLLGEEDGMPYWMNDGKRRLLEAEPSAPEFKPNVTVAADGSG
DFKTIKEALAKVPPKSASMYVMYIKAGTYKEYVSVGRPITNLVVIGDGDD
KTIITGNKNFKMNITTKDTATMGGDRERLLHEGREGGEHGGGREPPGRGA
ADTLYTHAQRQFFRDCTITGTIDFIFGNSQVVIQNCLILPRKPMDNQLNI
ITAQGRREKRSVGGTVMHNNTIEPHPDFKDSTGKIKTYLARPWKEYSRTI
YIQNEIGAFIDPKGWLEWNGDFALETLFYAEVENTGPGADMSQRAKWGGI
KTVTYADAQKEYTVEAFIQGEQFIPKYGVPFIPGLLP

>*Triticum aestivum* cultivar Chinese *WheatPME1-D* gene

ATGAGTAAAGGTGCGATCATCGGCGCGTCGACCGTCCTGGTGGTGGCGGT
CGTCGCCGCCGTGTGCGTCGTGTCCTTCAAGGGCAACGGCGGCGACAAGG
GCGATGGGGAGCTCACCACCTCCGTCAAGTCCGTCAAGGCCTTCTGCCAG
CCCATGGACTACAAGGAGACGTGCGAGGCGGAGCTGACCAAGGTGGGCGG
CAACGCCACGTCGCCGACGGAGCTCGCCAAGGCCATCTTCGAGGTGACCT
CGGAGAAGATCAGGAAGGCCATCAGCGAGTCGGCCACCCTGGAGGAGCTC
AAGAGCGACCCGCGCACGTCGGGGGCCCTCGAGAACTGCAAGGAGCTGCT
GGAGTACGCCATCGAGGACCTCAAGACCACCTTCGACCGCCTCGGCGGCT
TCGAGATGACCGACTTCAACAAGGCCGCCGCCGACCTCAAGACCTGGCTC
AGCGCCGCGCTCACCTACCAGGAGACCTGCCTCGACGGCTTCGCCAACAC
CACCACCGACGCCGCCGCCAAGATGCGCGGCGCGCTCAACGCCTCCCAGG
AGCTCACCGAGGACATCCTCGCCGTGGTCGACCAGTTCTCCGCCTCCCTC
GGCAGCCTCAACATCGGGAAGAGGAGGCTGCTAGGGGAGGAGGACGGCAT
GCCTTACTGGATGAACGACGGGAAGAGGCGGCTGCTGGAGGCCGAGCCCT
CCGCACCCGAGTTCAAGCCCAACGTCACCGTCGCCGCTGACGGCAGCGGC
GACTTCAAGACCATCAAGGAGGCGCTCGCAAAGGTGCCGCCCAAGAGCGC
CTCCATGTACGTCATGTACATCAAGGCCGGCACCTACAAGGAGTACGTCT
CCGTGGGCCGCCCTATCACCAACCTCGTCGTCATCGGCGACGGCGACGAC
AAGACCATCATCACCGGCAACAAGAACTTCAAGATGAACATCACCACCAA
GGACACCGCAACCATGGGAGGCAATCGGGAACGGCTTCTTCATGAAGGGC
GTGAGGGTGGAGAACACGGCGGGGGCCGAGAACCACCAGGCCGTGGCACT
GCGGGTGCAGAGCGACCAGGCCGTCTTCTACCAGTGCTACTTCGACGGGT
ACCAAGACACGCTCTACACCCACGCGCAACGGCAGTTCTTCCGCGACTGC
ACCGTCACCGGCACCATCGACTTCATCTTCGGCAACTCCCAGGTGGTCAT
CCAGAACTGCCTCATCCTGCCGCGCAAGCCCATGGACAACCAGCTCAACA
TCATCACCGCGCAAGGGCGGCGCGAGAAGCGCTCCGTCGGGGGCACCGTC
ATGCACAACAACACCATCGAGCCGCACCCGGACTTCAAGGACTCGACGGG
CAAGATCAAGACCTACCTGGCGCGGCCATGGAAGGAGTACTCTAGGACCA
TCTACATCCAGAACGAGATCGGCGCCTTCATCGACCCCAAGGGCTGGCTC
GAGTGGAACGGCGACTTCGCCCTCGAGACCCTCTTCTACGCCGAGGTGGA
GAACACCGGGCCCGGCGCCGATATGAGCCAGCGCGCCAAGTGGGGCGGCA
TCAAGACTGTCACCTACGCTGACGCACAAAAGGAGTACACCGTCGAGGCC
TTCATCCAGGGCGAGCAGTTCATCCCCAAGTACGGCGTGCCCTACATCCC
GGGGCTCCTCCCGCAGTCGGAGGCGGGGAGGG

>*Triticum aestivum* cultivar Chinese *WheatPME1-D* predicted protein

MSKGAIIGASTVLVVAVVAAVCVVSFKGNGGDKGDGELTTSVKSVKAFCQ
PMDYKETCEAELTKVGGNATSPTELAKAIFEVTSEKIRKAISESATLEEL
KSDPRTSGALENCKELLEYAIEDLKTTFDRLGGFEMTDFNKAAADLKTWL
SAALTYQETCLDGFANTTTDAAAKMRGALNASQELTEDILAVVDQFSASL
GSLNIGKRRLLGEEDGMPYWMNDGKRRLLEAEPSAPEFKPNVTVAADGSG
DFKTIKEALAKVPPKSASMYVMYIKAGTYKEYVSVGRPITNLVVIGDGDD
KTIITGNKNFKMNITTKDTATMGGNRERLLHEGREGGEHGGGREPPGRGT
ADTLYTHAQRQFFRDCTVTGTIDFIFGNSQVVIQNCLILPRKPMDNQLNI
ITAQGRREKRSVGGTVMHNNTIEPHPDFKDSTGKIKTYLARPWKEYSRTI
YIQNEIGAFIDPKGWLEWNGDFALETLFYAEVENTGPGADMSQRAKWGGI
KTVTYADAQKEYTVEAFIQGEQFIPKYGVPYIPGLLP

Figure S1. *WheatPME1* gene and protein sequences. Fasta sequences of hexaploid cv Chinese Spring *WheatPME1-A*, *WheatPME1*-B and *WheatPME1-D* genes and encoded polypeptides.
