# Supplementary material for: Cell wall traits as potential resources to improve resistance of durum wheat against Fusarium graminearum
Source: BMC Plant Biol. 2015 Jan 19;15:6. doi: 10.1186/s12870-014-0369-1 (PMC4298115; doi:10.1186/s12870-014-0369-1)
Supplement: Additional file 2: Figure S2. — Multiple alignment of WheatPME1 genes identified in Triticum aestivum, cv. Chinese Spring in the corresponding A, B and D genomes. In yellow are highlighted the SNPs between the A/D and B genomes. [file 12870_2014_369_MOESM2_ESM.pdf]

```
WheatPME1-A        ATGAGTAAAGGTGCGATCATCGGCGCGTCGACCGTCCTGGTGGTGGCGGT 50
WheatPME1-D        ATGAGTAAAGGTGCGATCATCGGCGCGTCGACCGTCCTGGTGGTGGCGGT 50
WheatPME1-B        ATGAGCAAAGGCGCCATAATCGGCGCGTCGACCGTCCTGGTGGTGGCGGT 50
                   ***** ***** ** ** ********************************

WheatPME1-A        CGTCGCCGCCGTGTGCGTCGTGTCCTTCAAGGGCAACGGCGGCGACAAGG 100
WheatPME1-D        CGTCGCCGCCGTGTGCGTCGTGTCCTTCAAGGGCAACGGCGGCGACAAGG 100
WheatPME1-B        CGTCGCCGCCGTCTGCGTCGTGTCCTTCAAGGGCAACGGCGGCGACAAGG 100
                   ************ *************************************

WheatPME1-A        GCGATGGGGAGCTCACCACCTCCGTCAAGTCCGTCAAGGCCTTCTGCCAG 150
WheatPME1-D        GCGATGGGGAGCTCACCACCTCCGTCAAGTCCGTCAAGGCCTTCTGCCAG 150
WheatPME1-B        GCGATGGGGAGCTCACCACCTCCGTCAAGTCCGTCAAGGCCTTCTGCCAG 150
                   *************************************************

WheatPME1-A        CCCATGGACTACAAGGAGACGTGCGAGGCGGAGCTGACCAAGGTGGGCGG 200
WheatPME1-D        CCCATGGACTACAAGGAGACGTGCGAGGCGGAGCTGACCAAGGTGGGCGG 200
WheatPME1-B        CCCATGGACTACAAGGAGACGTGCGAGGCGGAGCTGACCAAGGTGGGCGG 200
                   **************************************************

WheatPME1-A        CAACGCCACGTCGCCGACGGAGCTCGCCAAGGCCATCTTCGAGGTGACCT 250
WheatPME1-D        CAACGCCACGTCGCCGACGGAGCTCGCCAAGGCCATCTTCGAGGTGACCT 250
WheatPME1-B        CAACGCCACGTCGCCGACGGAGCTCGCCAAGGCCATCTTCGAGGTCACCT 250
                   ********************************************* ****

WheatPME1-A        CGGAGAAGATCAGGAAGGCCATCAGCGAGTCGGCCACCCTGGAGGAGCTC 300
WheatPME1-D        CGGAGAAGATCAGGAAGGCCATCAGCGAGTCGGCCACCCTGGAGGAGCTC 300
WheatPME1-B        CGGAGAAGATCAGGAAGGCCATCAGCGAGTCGGCCACCCTGGAGGAGCTC 300
                   **************************************************

WheatPME1-A        AAGAGCGACCCGCGCACGTCGGGGGCCCTCGAGAACTGCAAGGAGCTGCT 350
WheatPME1-D        AAGAGCGACCCGCGCACGTCGGGGGCCCTCGAGAACTGCAAGGAGCTGCT 350
WheatPME1-B        AAGAGCGACCCGCGCACGTCGGGGGCCCTCGAGAACTGCAAGGAGCTGCT 350
                   **************************************************

WheatPME1-A        GGAGTACGCCATCGAGGACCTCAAGACCACCTTCGACCGCCTCGGCGGCT 400
WheatPME1-D        GGAGTACGCCATCGAGGACCTCAAGACCACCTTCGACCGCCTCGGCGGCT 400
WheatPME1-B        GGAGTACGCCATCGAGGACCTCAAGACCACCTTCGACCGCCTCGGCGGCT 400
                   **************************************************

WheatPME1-A        TCGAGATGACCGACTTCAACAAGGCCGCCGCCGACCTCAAGACCTGGCTC 450
WheatPME1-D        TCGAGATGACCGACTTCAACAAGGCCGCCGCCGACCTCAAGACCTGGCTC 450
WheatPME1-B        TCGAGATGACCGACTTCAACAAGGCCGCCGCCGACCTCAAGACCTGGCTC 450
                   **************************************************

WheatPME1-A        AGCGCCGCGCTCACCTACCAGGAGACCTGCCTCGACGGCTTCGCCAACAC 500
WheatPME1-D        AGCGCCGCGCTCACCTACCAGGAGACCTGCCTCGACGGCTTCGCCAACAC 500
WheatPME1-B        AGCGCCGCGCTCACCTACCAGGAGACCTGCCTCGACGGCTTCGCCAACAC 500
                   **************************************************

WheatPME1-A        CACCACCGACGCCGCCGCCAAGATGCGCGGCGCGCTCAACGCCTCCCAGG 550
WheatPME1-D        CACCACCGACGCCGCCGCCAAGATGCGCGGCGCGCTCAACGCCTCCCAGG 550
WheatPME1-B        CACCACCGACGCCGCCGCCAAGATGCGCGGCGCGCTCAACGCCTCCCAGG 550
                   **************************************************

WheatPME1-A        AGCTCACCGAGGACATCCTCGCCGTGGTCGACCAGTTCTCCGCCTCCCTC 600
WheatPME1-D        AGCTCACCGAGGACATCCTCGCCGTGGTCGACCAGTTCTCCGCCTCCCTC 600
WheatPME1-B        AGCTCACCGAGGACATCCTCGCCGTGGTCGACCAGTTCTCCGCCTCCCTC 600
                   **************************************************

WheatPME1-A        GGCAGCCTCAACATCGGGAAGAGGAGGCTGCTAGGGGAGGAGGACGGCAT 648
WheatPME1-D        GGCAGCCTCAACATCGGGAAGAGGAGGCTGCTAGGGGAGGAGGACGGCAT 648
WheatPME1-B        GGCAGCCTCAACATCGGGAAGAGGAGGCTGCTAGGGGAGGAGGACGGCAT 650
                   ******************************  ******************

WheatPME1-A        GCCTTACTGGATGAACGACGGGAAGAGGCGGCTGCTGGAGGCCGAGCCCT 698
WheatPME1-D        GCCTTACTGGATGAACGACGGGAAGAGGCGGCTGCTGGAGGCCGAGCCCT 698
WheatPME1-B        GCCTTACTGGATGAACGACGGGAAGAGGCGGCTGCTGGAGGCCGAGCCCT 700
                   **************************************************
```

```
WheatPME1-A          CCGCACCCGAGTTCAAGCCCAACGTCACCGTCGCCGCTGACGGCAGCGGC 748
WheatPME1-D          CCGCACCCGAGTTCAAGCCCAACGTCACCGTCGCCGCTGACGGCAGCGGC 748
WheatPME1-B          CCGCACCGGAGTTCAAGCCCAACGTCACCGTCGCCGCTGACGGCAGCGGC 750
                     ******* ******************************************

WheatPME1-A          GACTTCAAGACCATCAAGGAGGCGCTCGCAAAGGTGCCGCCCAAGAGCGC 798
WheatPME1-D          GACTTCAAGACCATCAAGGAGGCGCTCGCAAAGGTGCCGCCCAAGAGCGC 798
WheatPME1-B          GACTTCAAGACCATCAAGGAGGCGCTCGCAAAGGTGCCGCCCAAGAGCGC 800
                     **************************************************

WheatPME1-A          CTCCATGTACGTCATGTACATCAAGGCCGGCACCTACAAGGAGTACGTCT 848
WheatPME1-D          CTCCATGTACGTCATGTACATCAAGGCCGGCACCTACAAGGAGTACGTCT 848
WheatPME1-B          CTCCATGTACGTCATGTACATCAAGGCCGGCACCTACAAGGAGTACGTCT 850
                     **************************************************

WheatPME1-A          CCGTGGGCCGCCCTATCACCAACCTCGTCGTCATCGGCGACGGCGACGAC 898
WheatPME1-D          CCGTGGGCCGCCCTATCACCAACCTCGTCGTCATCGGCGACGGCGACGAC 898
WheatPME1-B          CCGTGGGCCGCCCTATCACCAACCTCGTCGTCATCGGCGACGGCGACGAC 900
                     **************************************************

WheatPME1-A          AAGACCATCATCACCGGCAACAAGAACTTCAAGATGAACATCACCACCAA 948
WheatPME1-D          AAGACCATCATCACCGGCAACAAGAACTTCAAGATGAACATCACCACCAA 948
WheatPME1-B          AAGACCATCATCACCGGCAACAAGAACTTCAAGATGAACATCACCACCAA 950
                     **************************************************

WheatPME1-A          GGACACCGCAACCATGGGAGGCAATCGGGAACGGCTTCTTCATGAAGGGC 998
WheatPME1-D          GGACACCGCAACCATGGGAGGCAATCGGGAACGGCTTCTTCATGAAGGGC 998
WheatPME1-B          AGACACCGCAACCATGGGAGGCGATCGGGAACGGCTTCTTCATGAAGGGC 1000
                      ********************* ***************************

WheatPME1-A          GTGAGGGTGGAGAACACGGCGGGGGCCGAGAACCACCAGGCCGTGGCACT 1048
WheatPME1-D          GTGAGGGTGGAGAACACGGCGGGGGCCGAGAACCACCAGGCCGTGGCACT 1048
WheatPME1-B          GTGAGGGTGGAGAACACGGCGGGGGCCGAGAACCACCAGGCCGTGGCGCT 1050
                     *********************************************** **

WheatPME1-A          GCGGGTGCAGAGCGACCAGGCCGTCTTCTACCAGTGCTACTTCGACGGGT 1098
WheatPME1-D          GCGGGTGCAGAGCGACCAGGCCGTCTTCTACCAGTGCTACTTCGACGGGT 1098
WheatPME1-B          GCGGGTGCAGAGCGACCAGGCCGTCTTCTACCAGTGCTACTTCGACGGGT 1100
                     **************************************************

WheatPME1-A          ACCAAGACACGCTCTACACCCACGCGCAACGGCAGTTCTTCCGCGACTGC 1148
WheatPME1-D          ACCAAGACACGCTCTACACCCACGCGCAACGGCAGTTCTTCCGCGACTGC 1148
WheatPME1-B          ACCAAGACACGCTCTACACCCACGCGCAACGGCAGTTCTTCCGCGACTGC 1150
                     **************************************************

WheatPME1-A          ACCGTCACCGGCACCATCGACTTCATCTTCGGCAACTCCCAGGTGGTCAT 1198
WheatPME1-D          ACCGTCACCGGCACCATCGACTTCATCTTCGGCAACTCCCAGGTGGTCAT 1198
WheatPME1-B          ACCATCACCGGCACCATCGACTTCATCTTCGGCAACTCCCAGGTGGTCAT 1200
                     *** **********************************************

WheatPME1-A          CCAGAACTGCCTCATCCTGCCGCGCAAGCCCATGGACAACCAGCTCAACA 1248
WheatPME1-D          CCAGAACTGCCTCATCCTGCCGCGCAAGCCCATGGACAACCAGCTCAACA 1248
WheatPME1-B          CCAGAACTGCCTCATCCTGCCGCGCAAGCCCATGGACAACCAGCTCAACA 1250
                     **************************************************

WheatPME1-A          TCATCACCGCGCAAGGGCGGCGCGAGAAGCGCTCCGTCGGGGGCACCGTC 1298
WheatPME1-D          TCATCACCGCGCAAGGGCGGCGCGAGAAGCGCTCCGTCGGGGGCACCGTC 1298
WheatPME1-B          TCATCACCGCGCAAGGGCGCCGCGAGAAGCGCTCCGTCGGGGGCACCGTC 1300
                     ******************* ******************************

WheatPME1-A          ATGCACAACAACACCATCGAGCCGCACCCGGACTTCAAGGACTCGACGGG 1348
WheatPME1-D          ATGCACAACAACACCATCGAGCCGCACCCGGACTTCAAGGACTCGACGGG 1348
WheatPME1-B          ATGCACAACAACACCATCGAGCCGCACCCCGACTTCAAGGACTCGACGGG 1350
                     ***************************** ********************

WheatPME1-A          CAAGATCAAGACCTACCTGGCGCGGCCATGGAAGGAGTACTCTAGGACCA 1398
WheatPME1-D          CAAGATCAAGACCTACCTGGCGCGGCCATGGAAGGAGTACTCTAGGACCA 1398
WheatPME1-B          CAAGATCAAGACCTACCTGGCGCGCCCATGGAAGGAGTACTCCAGGACCA 1400
                     ************************ ***************** *******
```

```
WheatPME1-A        TCTACATCCAGAACGAGATCGGCGCCTTCATCGACCCCAAGGGCTGGCTC 1448
WheatPME1-D        TCTACATCCAGAACGAGATCGGCGCCTTCATCGACCCCAAGGGCTGGCTC 1448
WheatPME1-B        TCTACATCCAGAACGAGATCGGCGCCTTCATCGACCCCAAGGGCTGGCTC 1450
                   **************************************************

WheatPME1-A        GAGTGGAACGGCGACTTCGCCCTCGAGACCCTCTTCTACGCCGAGGTGGA 1498
WheatPME1-D        GAGTGGAACGGCGACTTCGCCCTCGAGACCCTCTTCTACGCCGAGGTGGA 1498
WheatPME1-B        GAGTGGAACGGCGACTTCGCCCTCGAGACCCTCTTCTACGCCGAGGTGGA 1500
                   **************************************************

WheatPME1-A        GAACACCGGGCCCGGCGCCGATATGAGCCAGCGCGCCAAGTGGGGCGGCA 1548
WheatPME1-D        GAACACCGGGCCCGGCGCCGATATGAGCCAGCGCGCCAAGTGGGGCGGCA 1548
WheatPME1-B        GAACACCGGGCCCGGCGCCGACATGAGCCAGCGCGCCAAGTGGGGCGGCA 1550
                   ********************* ****************************

WheatPME1-A        TCAAGACTGTCACCTACGCTGACGCACAAAAGGAGTACACCGTCGAGGCC 1598
WheatPME1-D        TCAAGACTGTCACCTACGCTGACGCACAAAAGGAGTACACCGTCGAGGCC 1598
WheatPME1-B        TCAAGACTGTCACCTACGCCGACGCGCAGAAGGAGTACACCGTCGAGGCC 1600
                   ******************* ***** ** *********************

WheatPME1-A        TTCATCCAGGGCGAGCAGTTCATCCCCAAGTACGGCGTGCCCTACATCCC 1648
WheatPME1-D        TTCATCCAGGGCGAGCAGTTCATCCCCAAGTACGGCGTGCCCTACATCCC 1648
WheatPME1-B        TTCATCCAGGGCGAGCAGTTCATCCCCAAGTACGGCGTGCCCTTCATCCC 1650
                   ******************************************* ******

WheatPME1-A        GGGGCTCCTCCCGCAGTCGGAGGCGGGGAGGG 1680
WheatPME1-D        GGGGCTCCTCCCGCAGTCGGAGGCGGGGAGGG 1680
WheatPME1-B        GGGGCTCCTCCCGCAGTCGGAGGCGGGGAGGG 1682
                   ********************************
```

**Figure S2.** Multiple alignment of *WheatPME1* genes identified in *Triticum aestivum*, cv Chinese Spring in the corresponding A, B and D genomes. In yellow are highlighted the SNPs between the A/D and B genomes.
