# Supplementary material for: Cell wall traits as potential resources to improve resistance of durum wheat against Fusarium graminearum
Source: BMC Plant Biol. 2015 Jan 19;15:6. doi: 10.1186/s12870-014-0369-1 (PMC4298115; doi:10.1186/s12870-014-0369-1)
Supplement: Additional file 3: Figure S3. — Multiple alignment of WheatPME1 from A, B and D genomes of Triticum aestivum cv. Chinese Spring and from Brachypodium distachyon (BdPME1). The yellow box indicates the pro region, whereas the green box corresponds to the PME domain. The protein is reported in C terminus-N terminus orientation. [file 12870_2014_369_MOESM3_ESM.pdf]

```
WheatPME1-A     MSKGAIIGASTVLVVAVVAAVCVVSFKGNGGDKGDGE----LTTSVKSVKAFCQPMDYKE 56
WheatPME1-D     MSKGAIIGASTVLVVAVVAAVCVVSFKGNGGDKGDGE----LTTSVKSVKAFCQPMDYKE 56
WheatPME1-B     MSKGAIIGASTVLVVAVVAAVCVVSFKGNGGDKGDGE----LTTSVKSVKAFCQPMDYKE 56
BdPME1          MSKPAIIGASTVLVVAVVAAVCVVSSKNNNGGGGGGEEGGQLSTSVKSVKAFCQPMDYKE 60
                *** ********************* *.*.*. *.**    *:*****************

WheatPME1-A     TCEAELTKVGGN-ATSPTELAKAIFEVTSEKIRKAISESATLEELKSDPRTSGALENCKE 115
WheatPME1-D     TCEAELTKVGGN-ATSPTELAKAIFEVTSEKIRKAISESATLEELKSDPRTSGALENCKE 115
WheatPME1-B     TCEAELTKVGGN-ATSPTELAKAIFEVTSEKIRKAISESATLEELKSDPRTSGALENCKE 115
BdPME1          TCEAELSKMSGDKPTSPTELAKAIFEATSAKINKAVAESATLEELKNDKRTSGALQNCKE 120
                ******:*:.*:  .************.** **.**::*********.* ******:****

WheatPME1-A     LLEYAIEDLKTTFDRLGGFEMTDFNKAAADLKTWLSAALTYQETCLDGFANTTTDAAAKM 175
WheatPME1-D     LLEYAIEDLKTTFDRLGGFEMTDFNKAAADLKTWLSAALTYQETCLDGFANTTTDAAAKM 175
WheatPME1-B     LLEYAIEDLKTTFDRLGGFEMTDFNKAAADLKTWLSAALTYQETCLDGFANTTTDAAAKM 175
BdPME1          LLEYAVEDLKTSFDKLGGFEMTDFNKAVDDLKTWLSAALTYQETCLDGFLNTTGDASAKM 180
                *****:*****:**:************. ******************* *** **:***

WheatPME1-A     RGALNASQELTEDILAVVDQFSASLGSLNIGKRRLLGEEDGMPYWMNDGKRRLLEAEPSA 235
WheatPME1-D     RGALNASQELTEDILAVVDQFSASLGSLNIGKRRLLGEEDGMPYWMNDGKRRLLEAEPSA 235
WheatPME1-B     RGALNASQELTEDILAVVDQFSASLGSLNIGKRRLLGEEDGMPYWMNDGKRRLLEAEPSA 235
BdPME1          KGALNASQELTEDILAVVDQFSATLGSLSFGKRRLLAD-DGAPTWMTDGKRRLMEASAGA 239
                :*********************:****.:******.: ** * **.******:**...*

WheatPME1-A     P---------EFKPNVTVAADGSGDFKTIKEALAKVPPKSASMYVMYIKAGTYKEYVSVG 286
WheatPME1-D     P---------EFKPNVTVAADGSGDFKTIKEALAKVPPKSASMYVMYIKAGTYKEYVSVG 286
WheatPME1-B     P---------EFKPNVTVAADGSGDFKTIKEALAKVPPKSASMYVMYIKAGTYKEYVSVG 286
BdPME1          PSSSSSSSPMDFEPNVTVAADGSGDFKTINEALAKVPPKSTAMYVMYVKAGTYKEYVSVG 299
                *          :*:*****************:**********::*****:************

WheatPME1-A     RPITNLVVIGDGDDKTIITGNKNFKMNITTKDTATMGGNRE-RLLHEGREGGEHGGG--- 342
WheatPME1-D     RPITNLVVIGDGDDKTIITGNKNFKMNITTKDTATMGGNRE-RLLHEGREGGEHGGG--- 342
WheatPME1-B     RPITNLVVIGDGDDKTIITGNKNFKMNITTKDTATMGGDRE-RLLHEGREGGEHGGG--- 342
BdPME1          RPITNLVMIGDGAEKTIITGNKNFKMNLTTKDTATMEAIGNGFFMRDIRVENTAGAANHQ 359
                *******:**** :*************:********  .  :  :::: *  .  *..

WheatPME1-A     ----REPPG---------RGTADTLYTHAQRQFFRDCTVTGTIDFIFGNSQVVIQNCLIL 389
WheatPME1-D     ----REPPG---------RGTADTLYTHAQRQFFRDCTVTGTIDFIFGNSQVVIQNCLIL 389
WheatPME1-B     ----REPPG---------RGAADTLYTHAQRQFFRDCTITGTIDFIFGNSQVVIQNCLIL 389
BdPME1          AVALRVQSDQAVFFQCTFDGYQDTLYTHAQRQFFRDCRITGTIDFIFGNSQVVIQNCLIQ 419
                    *   ..            *   ************** :*******************

WheatPME1-A     PRKPMDNQLNIITAQGRREKRSVGGTVMHNNTIEPHPDFKDSTGKIKTYLARPWKEYSRT 449
WheatPME1-D     PRKPMDNQLNIITAQGRREKRSVGGTVMHNNTIEPHPDFKDSTGKIKTYLARPWKEYSRT 449
WheatPME1-B     PRKPMDNQLNIITAQGRREKRSVGGTVMHNNTIEPHPDFKDSTGKIKTYLARPWKEYSRT 449
BdPME1          PRKPMDNQVNIITAQGRREKRSVGGTVMHNNTIEPHPDFVSSTGKIATYLARPWKEYSRT 479
                ********:******************************  .***** *************

WheatPME1-A     IYIQNEIGAFIDPKGWLEWNGDFALETLFYAEVENTGPGADMSQRAKWGGIKTVTYADAQ 509
WheatPME1-D     IYIQNEIGAFIDPKGWLEWNGDFALETLFYAEVENTGPGADMSQRAKWGGIKTVTYADAQ 509
WheatPME1-B     IYIQNEIGAFIDPKGWLEWNGDFALETLFYAEVENTGPGADMSQRAKWGGIKTVTYADAQ 509
BdPME1          IYIQNNIGAFIDPKGWLEWNGNFGLETLFYAEVDNHGPGADMSKRAKWGGIKTVTYEDAQ 539
                *****:***************:*.*********:* *******:************ ***

WheatPME1-A     KEYTVEAFIQGEQFIPKYGVPYIPGLLP-------- 537
WheatPME1-D     KEYTVEAFIQGEQFIPKYGVPYIPGLLP-------- 537
WheatPME1-B     KEYTVEAFIQGEQFIPKYGVPFIPGLLP-------- 537
BdPME1          KEFTVETFIQGQQFIPKFGVPYIPGLLPQSEDGRAH 575
                **:***:****:*****:***:******
```

Figure S3. Multiple alignment of *Wheat*PME1 from A, B and D genomes of *Triticum aestivum* cv. Chinese Spring and from *Brachypodium distachyon (Bd*PME1*)*. The yellow box indicates the pro region, whereas the green box corresponds to the PME domain. The protein is reported in C terminus-N terminus orientation.
