# Supplementary material for: Cell wall traits as potential resources to improve resistance of durum wheat against Fusarium graminearum
Source: BMC Plant Biol. 2015 Jan 19;15:6. doi: 10.1186/s12870-014-0369-1 (PMC4298115; doi:10.1186/s12870-014-0369-1)
Supplement: Additional file 4: Table S1. — Primer pairs sequences for housekeeping and target genes. [file 12870_2014_369_MOESM4_ESM.pdf]

Additional table 1 Primer pairs sequences for housekeeping and target genes

Gene	Forward Primer	Reverse primer	Product length (bp)	Melt peak (°C)
CDC	CAAATACGCCATCAGGGAGAACATC	CGCTGCCGAAACCACGAGAC	227	86.5
RLI	CGATTCAGAGCAGCGTATTGTTG	AGTTGGTCGGGTCTCTTCTAAATG	242	80.0
ADP-RF	GACCACCATCCTCTACAAG	AGCAGCACAGCATCAC	276	84.5
WheatPME1	TACTTGGGGATGAACTGCTC	GAGATCGGCGCCTTCATC	150	80.0
βTUB2	TGCTGTTCTGGTCGATCTTG	CCCTGAAGGTGGTCACAGTT	170	82.0
